# Fe-Containing MOFs as Seeds for the Preparation of Highly Active Fe/Al-SBA-15 Catalysts in the N-Alkylation of Aniline

**DOI:** 10.3390/molecules24152695

**Published:** 2019-07-24

**Authors:** Sareena Mhadmhan, Maria Dolores Marquez-Medina, Antonio A. Romero, Prasert Reubroycharoen, Rafael Luque

**Affiliations:** 1 Program in Petrochemistry and Polymer Science, Faculty of Science, Chulalongkorn University, Bangkok 10330, Thailand; 2 Departamento de Quimica Organica, Facultad de Ciencias, Universidad de Cordoba, Campus de Rabanales, Edificio Marie Curie (C-3), Ctra Nnal IV-A, Km 396, E14014 Cordoba, Spain; 3 Department of Chemical Technology, Faculty of Science, Chulalongkorn University, Bangkok 10330, Thailand; 4 People’s Friendship University of Russia (RUDN University), 6 Miklukho-Maklaya str., Moscow 117198, Russia

**Keywords:** mechanochemistry, mesoporous aluminosilicate, metal organic frameworks, imine synthesis, N-alkylation of aniline

## Abstract

We have successfully incorporated iron species into mesoporous aluminosilicates (Al-SBA-15) using a simple mechanochemical milling method. The catalysts were characterized by nitrogen physisorption, inductively coupled plasma mass spectrometry (ICP-MS), pyridine (PY) and 2,6-dimethylpyridine (DMPY) pulse chromatography titration, powder X-ray diffraction (XRD), X-ray photoelectron spectroscopy (XPS) and scanning electron microscopy with energy-dispersive X-ray spectroscopy (SEM-EDX). The catalysts were tested in the N-alkylation reaction of aniline with benzyl alcohol for imine production. According to the results, the iron sources, acidity of catalyst and reaction conditions were important factors influencing the reaction. The catalyst showed excellent catalytic performance, achieving 97% of aniline conversion and 96% of imine selectivity under optimized conditions.

## 1. Introduction

N-alkylation of amines is an important reaction in organic chemistry for the synthesis of N-containing compounds, basic intermediates used in dyestuff synthesis, herbicides, synthetic rubbers, pharmaceuticals and insecticides [1,2,3,4]. Among these nitrogen compounds, secondary amine or imine are of high interest. Traditional methods for the production of nitrogen-alkyl/aryl amine involve the amination of primary amine and alkyl/aryl halides, with inherent drawbacks including side product formation and the use of toxic alkylating agents. In recent years, N-alkylation of amines with alcohols emerged as a more sustainable protocol [5], with alcohols having a low toxicity and being inexpensive by-products generated during the reaction being only water.

In general, homogeneous catalysts of metal complexes (i.e., Ru, Ir, Rh, Pt, Au, Ni, Cu, and Fe) have been used in amine alkylation with alcohols [6,7,8,9,10]. Nonetheless, homogeneous catalysts present a complicated separation of the reaction medium, making it difficult to isolate them from the obtained products.

Metal based heterogeneous catalysts reported so far (especially transition metals such as Fe [11], Ni [12] and Cu [13] catalysts) could overcome some drawbacks of homogeneous systems. However, the reported chemistries suffered from aggregation of active metal, low selectivity and poor yields of target products, as well as long reaction times. Thus, the development of highly active and stable non-noble metal heterogeneous catalysts for N-alkylation reaction still remains a significant challenge. Recently, metal organic frameworks (MOFs) have shown good catalytic performance in several organic reactions [14,15,16]. At a molecular level, the structure of MOFs can provide an enhanced dispersion of active species [15,17]. Rigid frameworks of MOFs could protect the active sites from degradation or aggregation leading to excellent activity. 

Aluminum-containing mesoporous silicas (Al-SBA-15) have received great attention due to their ordered structure and uniformed mesopore size, reasonably high surface area and good thermal stability [18,19,20]. There are many protocols for catalyst synthesis [19,20,21]. Among these methods, mechanochemistry recently emerged as a promising alternative synthetic method towards the design of advanced catalytic materials based on mesoporous materials [19,21,22,23]. The use of mechanochemistry allows us to obtain catalysts with control of active sites (preferentially deposited on the external surface of the support) without using solvents and reducing the number of stages in the synthesis of nanomaterials. All of these make the technique a more sustainable alternative to conventional syntheses. The synthesis of supported nanoparticles using this method has been previously reported by our research group [24,25,26,27,28], as well as other scientists [29] demonstrating the applicability and versatility for the production of nanomaterials with tunable sizes and shapes.

In this work, iron oxide nanoparticles were mechanochemically incorporated on Al-SBA15 materials employing FeCl_3_, Fe-MIL-53, Fe-MIL-88 and Fe-MIL-101 as iron precursors. The utilization of MOFs in low quantities as seeds to generate active species on mesoporous materials is an interesting novel concept that has been only very recently reported [23-24]. These catalysts were characterized and subsequently tested for imine production via N-alkylation of aniline with benzyl alcohol. The effects of reaction temperature, time, molar ratio of benzyl alcohol to aniline and basic additive were investigated.

## 2. Results and Discussion

### 2.1. Catalytic Characterization

**Figure 1:**
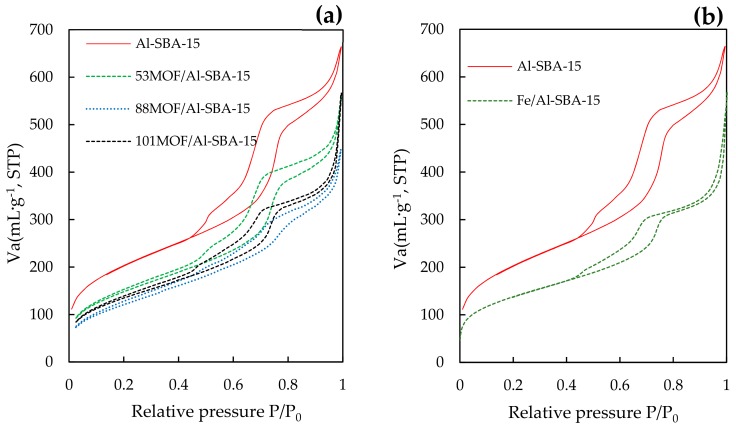
Nitrogen adsorption/desorption isotherms of (**a**) 53MOF/Al-SBA-15, 88MOF/Al-SBA-15 and 101MOF/Al-SBA-15 and (**b**) Fe/Al-SBA-15.

The catalysts were characterized by using various techniques including nitrogen physisorption, inductive coupling plasma mass spectrometry (ICP-MS), pyridine (PY) and 2,6-dimethyl pyridine (DMPY) pulse chromatography titration, X-ray diffraction (XRD), X-ray photoelectron spectroscopy (XPS) and scanning electron microscope-energy dispersive X-ray spectroscopy (SEM-EDX). The textural properties and porosity were determined by nitrogen adsorption/desorption isotherms. According to the international union of pure and applied chemistry (IUPAC), the nitrogen adsorption/desorption isotherms of all samples were of type IV with H1 type hysteresis loop between 0.5 and 0.8 P/P_0_ (Figure 1), characteristic of mesoporous materials [22,25]. The specific surface area, mean pore diameter, and pore volume of all samples are collected in Table 1. The surface area and average pore diameter decreased upon iron incorporation on the Al-SBA-15 materials in the mechanochemical step [18,26].

Surface acid properties were analyzed by using pyridine (PY, total acid sites) and 2,6-dimethylpyridine (DMPY, Brønsted sites) [19]. The total acidity of all catalysts increased as compared to parent Al-SBA-15 materials (Table 1), due to an increase in both Brønsted and mostly Lewis acidity in the catalysts, in good agreement with previously reported results [19,20,27,28].

The average iron content measured by ICP-MS of Fe/Al-SBA-15 catalyst was close to the theoretical value (1 weight percent (wt.%)) with the exception of that measured for 101MOF/Al-SBA-15 (see Table 1). However, the low iron content was sufficient to significantly improve the catalytic activity of all synthesized samples as compared to the parent Al-SBA-15 material.

**Figure 2:**
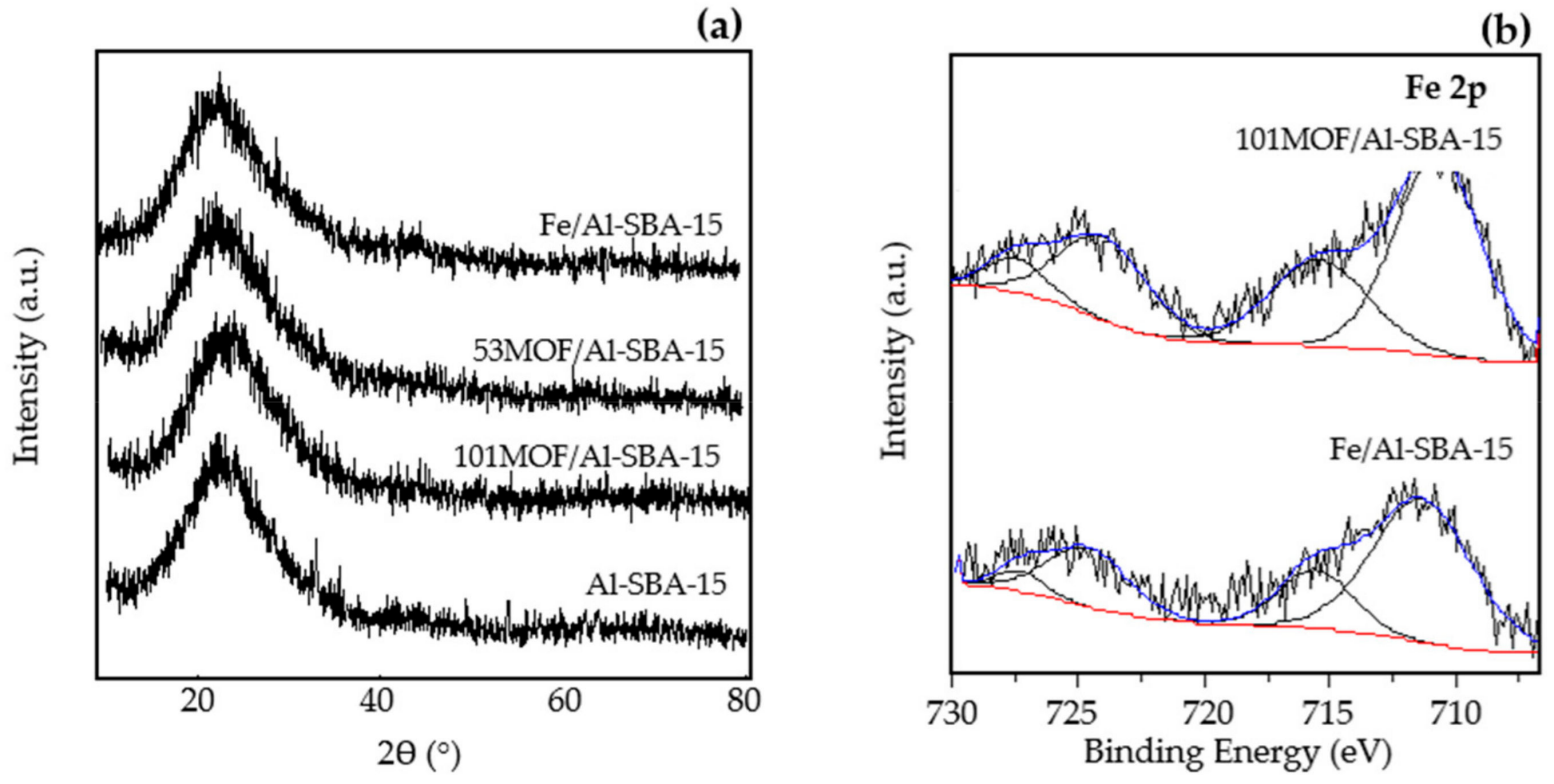
(**a**) Powder X-ray diffraction patterns of Al-SBA-15, 53MOF/Al-SBA-15, 101MOF/Al-SBA-15 and Fe/Al-SBA-15 and (**b**) XPS spectra of 101MOF/Al-SBA-15 and Fe/Al-SBA-15 in Fe 2p region.

Powder X-ray diffraction pattern of all samples are presented in Figure 2a. Iron incorporation on Al-SBA-15 materials could only be hinted in some materials, e.g., Fe/Al-SBA-15, due to the low amount of iron species on Al-SBA-15 [29]. Although no clear diffraction peaks of iron species could be visualized in XRD patterns, XPS spectra confirmed the presence of iron species by the peaks at 711.5 eV and 725 eV, corresponding to Fe 2p_3/2_ and Fe 2p_1/2_, respectively [30]. XPS results supported the presence of Fe^3+^ (mostly) and a small proportion of Fe^2+^ species as previously reported [31,32] (Figure 2b).

**Figure 3:**
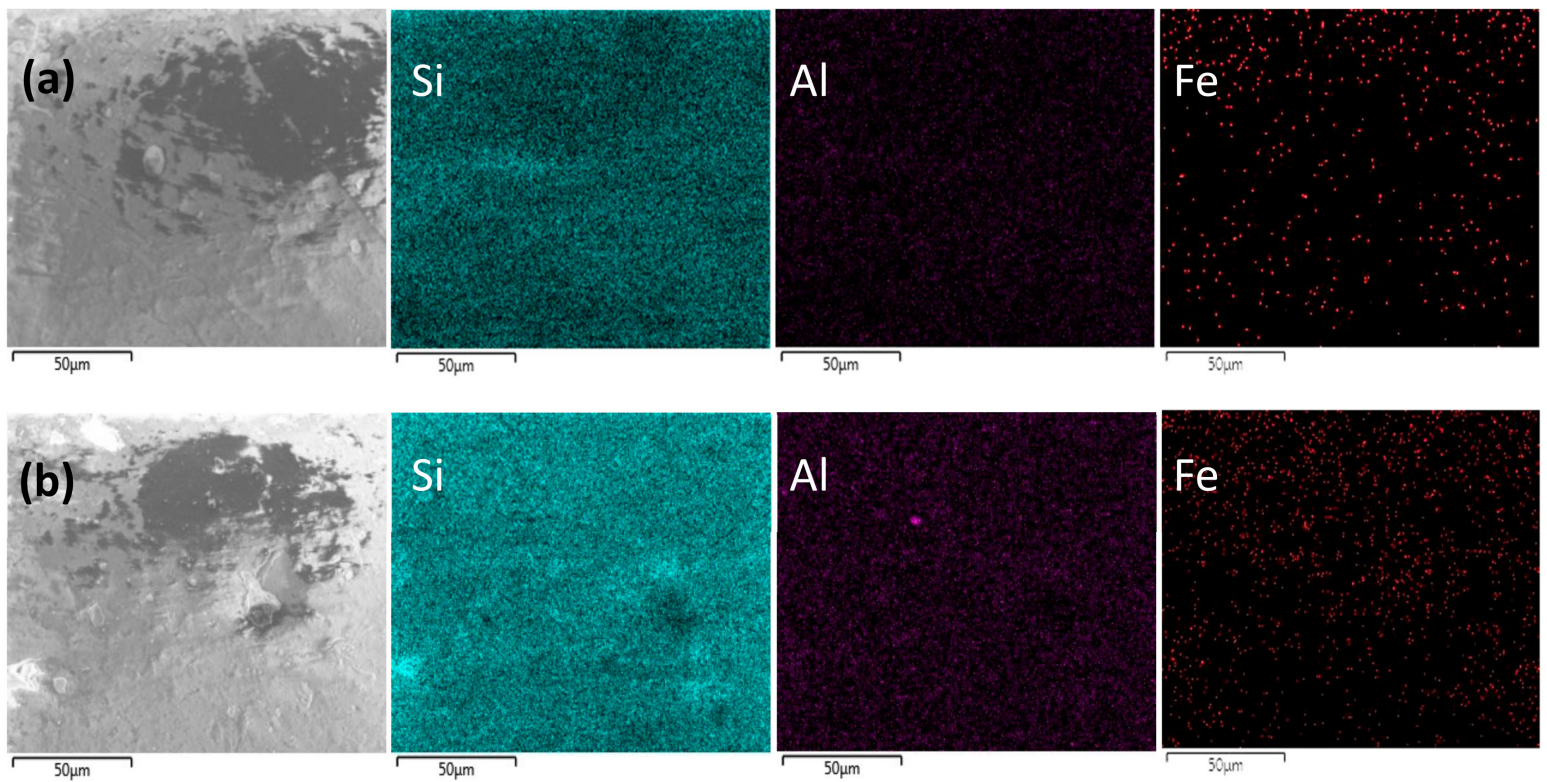
The SEM images for (**a**) Fe/Al-SBA-15 and (**b**) 101MOF/Al-SBA-15 and EDX elemental mapping profiles of the catalysts with Si (green), Al (purple) and Fe (red) distribution.

Scanning electron microscope-energy dispersive X-ray spectroscopy (SEM-EDX) with element mapping of the catalysts was carried out to investigate the iron oxide dispersion as presented in Figure 3. Elemental mapping confirmed the existence of Si, Al and Fe in the catalysts. Fe mapping images of Fe/Al-SBA-15 (Figure 3a) and 101MOF/Al-SBA-15 (Figure 3b demonstrated that iron species of 101MOF/Al-SBA-15 catalyst were comparably more homogeneously dispersed to that of Fe/Al-SBA-15. This result confirmed that the use of MOF as Fe seeds could enhance iron oxide nanoparticle dispersion on the surface of support.

### 2.2. Catalytic Performance

**Scheme 1:**
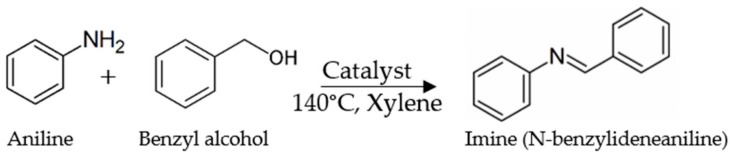
N-alkylation of aniline with benzyl alcohol.

The effect of various catalysts on the catalytic activity for N-alkylation of aniline with benzyl alcohol (Scheme 1) was compared in Table 2. Blank runs (both in the absence of catalyst and using Al-SBA-15) exhibited only a moderate conversion (ca. 50–60%) as compared to iron-based catalysts. Selectivity to the imine was good (typically over 70%), but both uncatalyzed and Al-SBA-15 catalyzed reactions gave rise to dibenzyl ether from the etherification of two molecules of benzyl alcohol. Comparing unsupported Fe and Al-SBA-15 supported Fe catalysts, it was found that both catalysts provided a good catalytic activity. However, the use of Al-SBA-15 as support was essential to provide an optimum catalytic performance. Among the different catalysts, 53MOF/Al-SBA-15 catalyst provided the highest catalytic activity under otherwise identical reaction conditions, achieving 94% conversion and 96% of imine selectivity.

Interestingly, the iron-containing catalyst synthesized using FeCl_3_ as precursor provided only comparable conversion/imine yield to the worst performing MOF/Al-SBA-15 material (containing three times less iron content, Table 1), indicating that iron incorporation was significantly more effective using MOFs as metal seeds to design high performing catalytic materials. The improved activities observed for MOF/Al-SBA-15 materials (despite their reduced iron content) are believed to be related to the highly dispersed and isolated iron oxide species present in the materials originated in the mechanochemical step due to the highly localized presence of Fe in the MOF structures employed as metal seeds. The acidity of catalyst significantly influenced the reaction, with an increase in aniline conversion also believed to be partially related to an increase in acidity. Additionally, but not least importantly, iron incorporation fully suppressed the competitive benzyl alcohol etherification under the investigated reaction conditions, providing almost quantitative selectivities to the imine product for all Fe-containing catalysts (Table 2).

**Figure 4:**
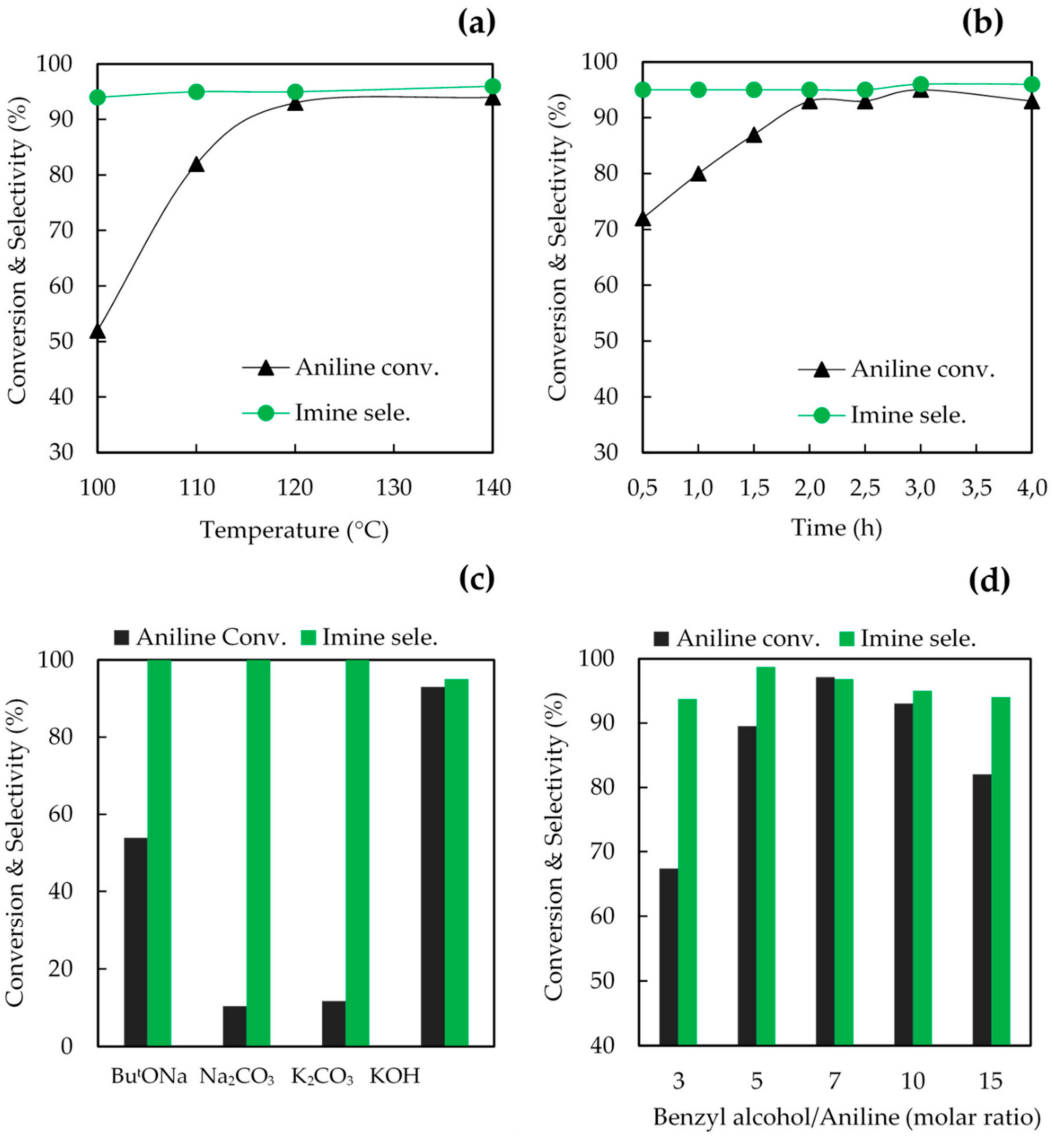
Catalytic activity over 53MOF/Al-SBA-15 catalyst, reaction conditions: 0.01 mol% catalyst (30 mg), 40.6 mmol xylene (5 mL) and (**a**) 1 mmol aniline (0.092 mL), 10 mmol benzyl alcohol (1.036 mL), 0.4 mmol KOH (20 mg) for 3 h, (**b**) 1 mmol aniline (0.092 mL), 10 mmol benzyl alcohol (1.036 mL), 0.4 mmol KOH (20 mg), at 120 °C, (**c**) 1 mmol aniline (0.092 mL), 10 mmol benzyl alcohol (1.036 mL), at 120 °C for 2 h and (**d**) 1 mmol aniline (0.092 mL), 0.4 mmol KOH (20 mg), at 120 °C for 2 h.

On the different catalysts, 53MOF/Al-SBA-15 provided the best results and was subsequently selected for further optimization. The optimum reaction temperature, time, molar ratio of benzyl alcohol to aniline and basic additive were studied. As seen in Figure 4a, aniline conversion rapidly increased from 52% to 93% at the temperature range of 100-120 °C. However, a further increase to 140 °C provided similar conversions in the systems (93–94%). Hence, the optimum temperature was found to be 120 °C. The effect of reaction time was then investigated, with conversions raising from 72% to 93% and approaching steady state conditions after 2 h (optimum reaction time, Figure 4b).

Various bases including Bu^t^ONa, Na_2_CO_3_, K_2_CO_3_ and KOH were subsequently studied to investigate the effect of the added base in the alkylation reaction. Results are presented in Figure 4c. Only 11% of aniline conversion was observed in the absence of base. In the presence of weak bases such as Bu^t^ONa, Na_2_CO_3_ and K_2_CO_3_, low to moderate conversions were found (10–54%). Comparably, the use of KOH provided optimum results (93% aniline conversion).

Additionally, studies on the effect of benzyl alcohol to aniline molar ratios were performed and results have been presented in Figure 4d. The molar ratio of benzyl alcohol to aniline played an important role for the reaction. Among all of them, the reaction system containing benzyl alcohol and aniline in a molar ratio of 1:7 provided the best activity, achieving the highest aniline conversion ca. 97%, 96% imine selectivity. Higher benzyl alcohol/aniline ratios (ca. 1:10 and 1:15 molar ratio) led to a slightly decreased aniline conversion due to the formation of benzyl oligomers (detected by gas chromatography-mass spectrometry (GC-MS)) that could be adsorbed on the surface of the catalyst, partially blocking the active sites (fouling). Optimum conditions for aniline alkylation employing the best performing 53MOF/Al-SBA-15 catalyst were found to be 1 mmol aniline (0.092 mL), 7 mmol benzyl alcohol (0.725 mL), xylene (5 mL), catalysts (30 mg), KOH (20 mg), temperature 120 °C and 2 h of reaction time.

**Figure 5:**
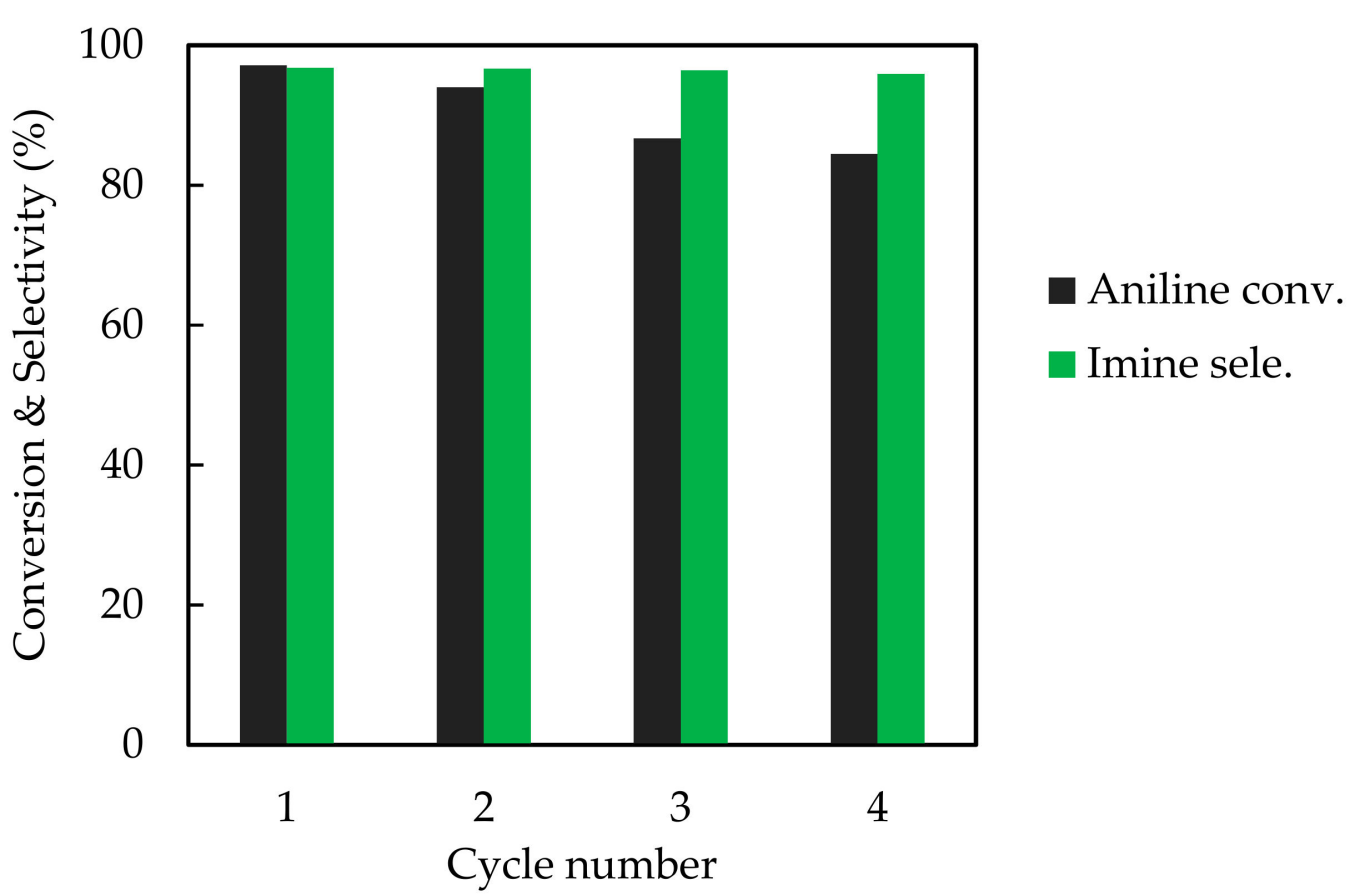
Reusability of 53MOF/Al-SBA-15 catalyst in the N-alkylation of aniline with benzyl alcohol. Reaction conditions: 1 mmol of aniline (0.092 mL), 7 mmol of benzyl alcohol (0.725 mL), 40.6 mmol xylene (5 mL), 0.01 mol% catalysts (30 mg), 0.4 mmol KOH (20 mg), temperature 120 °C, 2 h.

The reusability of the 53MOF/Al-SBA-15 catalyst was eventually studied under optimum reaction conditions. For each recycle, the spent catalyst was recovered from the reaction mixture by filtration, followed by thorough washing and drying at 100 °C for 1 h prior to its reuse in the next run. Results presented in Figure 5 pointed to a rather stable aniline conversion and imine selectivity upon reuses, which further supported the excellent recyclability and stability of the mechanochemically synthesized catalysts in good agreement with previous reports from the group, indicating that 53MOF/Al-SBA-15 could well resist leaching and deactivation of active sites.

## 3. Materials and Methods

### 3.1. Catalyst Preparation

#### 3.1.1. Synthesis of Al-SBA-15

The preparation of the mesoporous aluminosilicate (Al-SBA-15) was carried out according to a procedure reported by Ojeda et al. [33]. The molar ratio of silicon to aluminum was 20. Typically, pluronic P123 tri-block copolymer (Sigma-Aldrich, Madrid, Spain) (20 g) was dissolved in 750 mL of HCl solution (Panreac, Barcelona, Spain) (0.2 M, pH 1.5) for 2 h at 40 °C under stirring. Then, tetraethyl orthosilicate (TEOS) (Sigma-Aldrich) (25 mmol) and aluminum isopropoxide (Sigma-Aldrich) (10 mmol) were added into the mixture solution and then stirred at 40 °C for 24 h. After that, the mixture solution was transferred to 100 mL autoclave and kept for 48 h at 100 °C. Finally, the obtained material was filtered, dried at 60 °C overnight and calcined at 550 °C for 8 h.

#### 3.1.2. Synthesis of Catalysts

Iron was incorporated on Al-SBA-15 materials by using mechanochemical planetary ball milling method with a different iron source such as FeCl_3_ (Fe), Sigma-Aldrich, as well as pre-synthesized Fe-MIL-53 (53MOF), Fe-MIL-88 (88MOF) and Fe-MIL-101 (101MOF) as metal seeds. Fe-MIL-53, Fe-MIL-88 and Fe-MIL-101 were synthesized by using previously reported protocols [34]. Typically, 2 g of Al-SBA-15 and 1 %wt. of desired iron precursor were introduced into the planetary ball mill jar (125 mL) containing 18 stainless steel balls (10 mm, 4 g each ball). The mixture was ground at 350 rpm for 10 min (optimum conditions) [19,21]. The material was calcined at 550 °C for 4 h. The materials obtained were identified as Fe/Al-SBA-15, 53MOF/Al-SBA-15, 88MOF/Al-SBA-15 and 101MOF/Al-SBA-15, respectively.

### 3.2. Catalyst Characterization

The catalysts were characterized by several techniques, including nitrogen physisorption, inductive coupling plasma mass spectrometry (ICP-MS), X-ray diffraction (XRD), X-ray photoelectron spectroscopy (XPS) and scanning electron microscope-energy dispersive X-ray spectroscopy (SEM-EDX).

The nitrogen sorption isotherms were determined by using the micromeritics automatic analyzer ASAP 2000 (Micromeritics Instrument Corp., Norcross, GA, USA). Firstly, each sample was degassed at 130 °C overnight under vacuum (*p* < 10^‒2^ Pa). The linear determination of the Brunauer-Emmett-Telller (BET) equation was carried out to obtain specific surface areas.

Inductively coupled plasma mass spectrometry (ICP-MS) was used for quantitative metal analysis of the catalysts, using an Elan DRC-e ICP-MS (PerkinElmer SCIEX, Billerica, MA, USA) located in the Central Service of Research Support (SCAI) at Universidad de Cordoba, Spain.

Surface acid properties were evaluated using pyridine (PY) which analyzed total acidity, and 2,6-dimethyl pyridine (DMPY) that interact only with the Brønsted acid sites, at 250 °C. The pulses were carried out by means of a microinjector in the catalytic bed from a cyclohexane solution of the titrant (0.989 M in PY and 0.956 M in DMPY). The catalyst was standardized at each titration in a dehydrated and deoxygenated nitrogen flow (50 mL∙min^‒1^) (99.999% purity) at 250 °C. The catalyst used (~0.03 g) was fixed by means of Pyrex glass wool stoppers, inside a stainless-steel tubular microreactor of 4 mm internal diameter. The injected base was analyzed by gas chromatography with a flame ionization detector (FID), using an analytical column 0.5 m in length, containing 5% by weight of polyphenylether in Chromosorb AW-MCS 80/100 (Supelco Analytical, Bellefonte, PA, USA).

The catalyst crystallinity was evaluated by using powder X-ray diffraction analysis with a Bruker D8-Advanced Diffractometer (40 kV, 40 mA) and Cu X-ray tube (λ = 0.15 A) (Bruker AXS, Karlsruhe, Germany). X-ray diffraction (XRD) patterns were measured in the range of 10–80° (2ϴ), with 0.02° step sizeat 20 s of counting time per step.

X-ray photoelectron spectroscopy (XPS) measurements were performed by using an ultra-high vacuum (UHV) multipurpose surface analysis system Specs^TM^ with the Phoibos 150-MCD energy detector. The experiments was analyzed at pressures <10^‒10^ mbar by using a conventional X-ray source (XR-50, Specs, Berlin, Germany), Mg-Kα, hv = 1253.6 eV, 1 eV = 1.603 × 10^‒19^ J) in a stop and go mode. The deconvolution curves and the quantification of the components were obtained by using the XPS CASA program (Casa Software Ltd., Cheshire, UK).

The elemental analysis of synthesized catalysts was investigated by using a JEOL JSM 7800F scanning electron microscope (JEOL Ltd., Akishima, Tokyo, Japan) equipped with an Inca Energy 250 microanalysis system, window detector of Si/Li type, detection range from boron to uranium and resolution range of 137–5.9 keV.

### 3.3. Reaction Testing

N-alkylation of aniline with benzyl alcohol was carried out using a parallel reaction system (Carrusel Reaction StationTM, Radleys Discovery Technologies Ltd., Saffron Walden, UK). Typically, 1 mmol aniline (0.092 mL, assay 99%), 7 mmol benzyl alcohol (0.725 mL, assay 99%), xylene (5 mL) as solvent, KOH (20 mg) as promoter and catalyst (30 mg) were mixed with magnetic stirring at 120 °C for 2 h. The sample was evaluated by GC (7890A)-MS (5975D inert MSD with triple-axis detector) equipped with capillary column HP-5MS (60 m × 0.32 mm). Moreover, the spent catalyst was recovered from the reaction mixture by filtration, washing and drying at 100 °C for 1 h. It was then reused in the next reaction.

## 4. Conclusions

The mechanochemical incorporation of iron species into Al-SBA-15 material using MOFs as metal seeds, namely MIL-53(Fe), MIL-88(Fe) and MIL-101(Fe), was successfully accomplished in this work, rendering highly active and stable Fe-containing catalysts for the N-alkylation of aniline with benzyl alcohol. The 53MOF/Al-SBA-15 catalyst exhibited excellent catalytic performance and stability, with quantitative imine yields under optimized conditions (97% conversion, 96% imine selectivity). Fe-containing materials were highly stable and could be reused successfully. These findings may offer great opportunities for improving imine synthesis using cheap and environmentally friendly iron oxide heterogeneous catalysts.

## Figures and Tables

**Table 1:** Textural and surface acid properties of synthesized materials.

Samples	S_BET_^a^ (m^2^·g^−1^)	D_p_^b^ (nm)	V_p_^c^ (mL·g^−1^)	Fe Content ^d^ (wt.%)	Acidity ^e^ (µmol·g^−1^)	
					PY	DMPY
Al-SBA-15	736	8.5	0.8	-	82	61
53MOF/Al-SBA-15	538	3.2	0.8	0.22/0.21 ^*^	220	124
88MOF/Al-SBA-15	475	3.0	0.7	0.18	219	72
101MOF/Al-SBA-15	490	3.7	0.8	0.30	94	51
Fe/Al-SBA-15	500	6.2	0.5	1.00	198	30

^a^ S_BET_: surface area was calculated by the Brunauer-Emmett-Teller (BET) equation. ^b^ D_p_: average pore size diameter was calculated by the Barret-Joyner-Halenda (BJH) equation. ^c^ V_p_: pore volumes were calculated by the Barret-Joyner-Halenda (BJH) equation. ^d^ Fe content as determined by ICP-MS.^*^ Fe content of spent catalyst after reuse as determined by SEM-EDX. ^e^ Catalyst acidity was analyzed by PY and DMPY pulse chromatography titration (250 °C).

**Table 2:** N-alkylation of aniline with benzyl alcohol by different catalysts.

Samples	Conversion (%)	Imine Select (%)	Imine Yield (%)
Blank (no catalyst)	54	76	41
Al-SBA-15	61	71	43
FeCl_3_	75	88	66
53MOF	77	82	63
88MOF	73	87	64
101MOF	78	80	62
53MOF/Al-SBA-15	94	96	90
88MOF/Al-SBA-15	90	94	85
101MOF/Al-SBA-15	85	93	79
Fe/Al-SBA-15	77	98	76

Reaction conditions: 1 mmol of aniline (0.092 mL), 10 mmol of benzyl alcohol (1.036 mL), 40.6 mmol xylene (5 mL), 0.01 mol% catalysts, 0.4 mmol KOH (20 mg), temperature 140 °C, 3 h. The remaining selectivity to 100 corresponds to benzyl dimers and oligomers derived from etherification reactions of benzyl alcohol.
